# Intra-Venous Injections in the 18th Century, and Earlier

**Published:** 1918-08

**Authors:** 


**Intra-Venous Injections in the 18th Century, and Earlier.** Le Progres Medical, Dec. 22, 1917, reproduces an illustration from Laurence Heister's Surgery, published 1750, showing, somewhat diagramatically, the veins of the elbow and anterior surface of the arm distended by a tourniquet, the left hand of the surgeon holding a trochar introduced into a vein, while the right hand is squeezing a bladder connected with the trochar. The method is, however, much older, Heister himself alluding to it as somewhat dangerous and fallen into desuetude, after having enjoyed a considerable vogue about 1660-1680, and being ascribed to Major or Wrenn. It seems to have been employed chiefly in apoplexy and dyspnoea, when deglutition was difficult or impossible, also to avoid the action of the digestive juices on medicaments. Leprosy, gout, epilepsy, consumption, scurvy, venereal disease (the various ones being strangely confused by most authors in the 17th and early 18th centuries) and malignant fevers are also mentioned. Purmann practiced the method successfully on himself but the general experience was that sudden death or stupidity, delirium or melancholy might develop and the French Senate officially condemned the use of intravenous medication. (Note: Aside from the more obvious factors connected with ignorance of asepsis, embolism, etc., it should be remembered that the success of this and many other procedures in medicine depends to a large degree upon the availability of rubber whose introduction lies within the memory of many persons still living. Ed.)

